# Management of a giant pediatric craniopharyngioma: staged endoscopic endonasal and cranial approaches

**DOI:** 10.3171/2026.7.FOCVID26136

**Published:** 2026-10-01

**Authors:** Akshay V. Save, Stella Chung, Carlos Pinheiro-Neto, Paul A. Gardner, Yosef M. Dastagirzada

**Affiliations:** 1 Department of Neurosurgery, NYU Langone Health, New York;; 2 Department of Oculoplastics, NYU Langone Health, New York; and; 3 Department of Otolaryngology, NYU Langone Health, New York, New York

**Keywords:** pediatric craniopharyngioma, endoscopic endonasal approach, eyebrow approach, supraorbital craniotomy, minimally invasive

## Abstract

This report presents a complex case of an 8-year-old female with a giant 8-cm adamantinomatous craniopharyngioma. She had a mixed solid/cystic, multicompartmental tumor with brainstem compression, vascular encasement, obstructive hydrocephalus, and acute stroke on presentation. After neurological improvement with an external ventricular drain, she underwent an extended endonasal approach followed by a left supraorbital craniotomy to address the residual lateral tumor. We achieved a near gross-total resection with improvement in visual acuity, neurocognitive function, and hemiparesis. This case demonstrates that staged skull base approaches can lead to clinically significant and safe resections in the pediatric population.

The video can be found here: https://stream.cadmore.media/r10.3171/2026.7.FOCVID26136

## Transcript

### 0:21

We present a complex case of a giant pediatric craniopharyngioma managed with staged endoscopic endonasal and cranial approaches.

### 0:29 Clinical Presentation.

Our patient is an 8-year-old girl with a history of adamantinomatous craniopharyngioma, presenting with progressive worsening of headaches, acute right-sided hemiparesis, and altered mental status. Pertinent history can be found below. CT scan demonstrates acute obstructive hydrocephalus secondary to a giant craniopharyngioma and a left parietal hypodensity concerning for an acute infarct. She emergently underwent EVD placement. Diffusion-weighted imaging confirmed multifocal left hemispheric infarcts. Contrast-enhanced imaging delineates a giant sellar and suprasellar mass with diffuse calcifications and heterogeneous enhancement. There is significant compression of the optic apparatus, frontal lobes, and brainstem. Comparative imaging demonstrates significant interval growth of both cystic and solid components since the time of her diagnosis. Diagnostic cerebral angiogram shows critical stenosis of the left > right supraclinoid ICA, an occluded left A1, and severely stenotic left PCA.

### 1:44 Clinical Course.

Her mental status and motor exam improved drastically over the course of 5 days. Updated ophthalmological evaluation was notable for severe decline in vision loss, resulting in 20/100 OD and no light perception OS. We discussed the recommendation with family for resection using a two-staged approach given the multicompartmental nature of the tumor, with the goals of surgery as detailed below.

### 2:06 Stage 1.

Stage 1 would include an expanded endoscopic endonasal approach for tumor resection. After initial exposure and harvest of the right-sided nasoseptal flap by our ENT team, a high-speed burr was used to drill the rostrum and nonpneumatized bone down to the face of the sella. It was expanded to identify the bilateral parasellar carotid arteries, medial optic canals, and dura of the planum, limbus sphenoidale, tuberculum, sella, and clivus.

### 2:40 Dural Opening.

The dura was initially opened below the superior intercavernous sinus, which was eventually coagulated and incised. The dura was incised toward the sphenoid limbus on either side, and tumor was internally debulked with two-suction technique. After entering the medial cavernous wall on the left and cutting the inferior hypophyseal artery, we had access to the posterior clinoid, which was removed with a combination of drilling and Kerrison rongeurs.

### 3:15 Pituitary Transposition.

The interclinoidal and caroticoclinoidal ligaments were coagulated and incised, allowing for complete hemitransposition of the pituitary gland. We then visualized the inferior pole of the tumor on the left and the membrane of Liliequist, which was protected with a patty. There was an excellent plane between the tumor capsule and arachnoid overlying the basilar and brainstem. Tumor dissection on the left revealed the midbrain, the oculomotor nerve, and a diminutive left PCA/SCA. We find the NICO Myriad system to be quite efficient at debulking tumors of this size and consistency. The left optic nerve was severely compressed and followed posteriorly. Dissection of the subfrontal cyst wall and tumor on the right with a 45° scope led us to the olfactory tract, as well as the anterior commissure and bilateral A2 segments of the ACAs. The tumor was severely compressing the optic chiasm with a nearly translucent left optic chiasm/nerve. This was dissected free with meticulous microdissection. The infrachiasmatic tumor was followed posteriorly toward the hypothalamus, which was sharply dissected off the tumor. As we followed the tumor posteriorly, we identified the third ventricle. Further tumor was delivered using angled dissectors, with care to preserve all surrounding vessels. The optic chiasm was significantly decompressed at the end of the resection, and you can appreciate residual tumor lateral to the left optic nerve. All major posterior circulation vessels were seen preserved and papaverine placed over them. Hemostasis was achieved with warm irrigation and Avitene sandwiches.

### 5:49 Button Graft Reconstruction.

Reconstruction was performed using a button graft technique with dura matrix inlay and fascia lata overlay followed by placement of the nasoseptal flap, which demonstrated brisk and bright perfusion with indocyanine green angiography.

### 6:25 Postoperative Course.

Postoperative day 1 she was noted to have worsening vision and MRI was notable for intradural extension of the flap. We brought her back to the OR emergently for revision, where papaverine was used once again, and the flap placed more superficially to avoid overt compression of the dramatically exposed optic nerves. A VP shunt was placed on postoperative day 9 given the inability to wean EVD. She was discharged home on postoperative day 18, at her neurological baseline with improved visual acuity (20/100 OD, light perception OS) and desmopressin added to her regimen.

### 6:58 Postoperative Imaging.

Postoperative MRI demonstrates significant resection of majority of the tumor with expected residual in the left opticocarotid cistern.

### 7:16 Stage 2.

Stage 2 included a left eyebrow incision and supraorbital craniotomy for resection of the residual tumor 6 weeks later. After sharp microdissection of the proximal sylvian fissure, we are able to identify the most anterior and lateral component of the tumor. Circumferential tumor dissection is performed, and the MCA bifurcation is identified. The left prechiasmatic optic nerve is identified as well. The encased left ICA is identified and skeletonized with microdissection and followed to the bifurcation where a severely stenotic A1 is seen. Tumor is resected around the encased vessel, preserving critical perforators. The tumor was densely adherent to the left optic nerve and A1 segment. A small remnant was left over the undersurface of A1. The Zeiss QEVO endoscope was used and helped identify a small residual anteriorly along the distorted optic nerve, which was subsequently resected. She was discharged postoperative day 4 at her neurological baseline. Immediate and 2-week postoperative pictures demonstrate an excellent cosmetic outcome.

### 9:05 Postoperative Imaging.

Postoperative imaging demonstrates no overt evidence of bulky residual tumor. Now, nearly a month out from her second-stage surgery, she is excelling in school with no new deficits. Visual acuity has improved to 20/70 OD with resolution of previous temporal field cut and intermittent light perception OS.

### 9:35 Discussion.

We demonstrate that giant craniopharyngiomas can be amenable to maximal safe resection using various skull base surgical techniques, even in the pediatric population.^1–3^ The expanded endoscopic endonasal approach allows for clinically significant decompression and improved visual acuity, due to the direct visualization of the interface between tumor and the optic apparatus.^4^ Consider flap positioning and microvascular spasm in cases of acute postoperative vision loss. Experienced skull base and pediatric skull base teams at a high-volume center are critical to improving outcomes in these complex cases.^5^

Thank you.

## Disclosures

Dr. Gardner reported consulting fees from Mizuho, Medtronic, and Sutter, and royalties from Peter Lazic, outside the submitted work.

## Author Contributions

Primary surgeon: Pinheiro-Neto, Gardner. Assistant surgeon: Save, Chung. Editing and drafting the video and abstract: Dastagirzada, Save. Critically revising the work: Dastagirzada, Save. Reviewed submitted version of the work: Dastagirzada, Save, Chung, Gardner. Approved the final version of the work on behalf of all authors: Dastagirzada.

## Supplemental Information

### Patient Informed Consent

The necessary patient informed consent was obtained in this study.
